# Supplementary material for: RNA sequencing in adult Drosophila females identifies estrogen-related receptor-dependent transcriptional changes in metabolism, DNA replication, and translation
Source: G3 (Bethesda). 2026 Jul 21;16(9):jkag186. doi: 10.1093/g3journal/jkag186 (PMC13535418; doi:10.1093/g3journal/jkag186)
Supplement: Supplementary file 1 [file jkag186_supplementary_data.zip › Supplemental_Table_Titles_G3-2026-406916.docx]

**Supplementary Materials for:**

**RNA Sequencing in Adult *Drosophila* Females Identifies Estrogen-Related Receptor-Dependent Transcriptional Changes in Metabolism, DNA Replication, and Translation**

**Sophie A. Fleck^1^, Eleanor B. Goldstone^1^, and Lesley N. Weaver^1*^**

^1^Department of Biology, Indiana University, Bloomington, IN 47405, USA.

***Corresponding author. Email: lnweaver@iu.edu**

**This file includes:**

Titles for Supplementary Tables.

**SUPPLEMENTARY TABLES**

**Supplementary Table 1. Total number of mapped reads for each *ERR* conditional knockout library.**

**Supplementary Table 2. List of all transcripts and their relative abundance in control and *ERR^cond.2^* females.**

**Supplementary Table 3. List of all transcripts and their relative abundance in control and *ERR^cond.19-4^* females.**

**Supplementary Table 4. PANGEA analysis of common downregulated genes in *ERR* conditional knockout females.**

**Supplementary Table 5. PANGEA analysis of common upregulated genes in *ERR* conditional knockout females.**

**Supplementary Table 6. Common and sex-specific genes downregulated in *ERR^cond.2^* conditional knockouts.**

**Supplementary Table 7. PANGEA analysis of common downregulated genes in *ERR^cond.2^* conditional knockout females and males.**

**Supplementary Table 8. PANGEA analysis of downregulated genes specifically in *ERR^cond.2^* females.**

**Supplementary Table 9. Common and sex-specific genes upregulated in *ERR^cond.2^* conditional knockouts.**

**Supplementary Table 10. PANGEA analysis of upregulated genes specifically in *ERR^cond.2^* females.**
